# Assessment of the views of intensive care nurses about the effect of patient visiting hours on patients and nursing care

**DOI:** 10.1186/2197-425X-3-S1-A922

**Published:** 2015-10-01

**Authors:** H Sütçü Çiçek, N Cambaztepe, B Kılıç

**Affiliations:** Gulhane Military Medical Academy, School of Nursing, Etlik, Turkey; Gulhane Military Medical Academy, Department of Norology, Etlik, Turkey

## Introduction

Policies of visiting in intensive care units effect both patients care and nursing care.

## Objective

This study aims to assess views of intensive care nurses about the effect of patient visiting hours on patients and nursing care.

## Methods

A data gathering questionnaire consisting of 20 questions has been used in this descriptive research. Among the nurses working in the departments of medical and surgical intensive care units of a teaching and research hospital, the ones who have given their consents has been included in the study.

## Results

120 nurses who are working at medical and surgical intensive care units have been included in the study. Mean age for the nurses is 29 ± 5.7 and 82.5% of them have bachelor's degrees. All the nurses participating in the study stated that patient visiting hours are not completely flexible and individual, age and time constrictions do exist. 88.3% of the nurses stated that they take isolation precautions for the visitors when the visitors are in the intensive care unit. 92.3% of the medical intensive care nurses and 52% of the surgical intensive care nurses see the patient visiting hours as a source of stress. Furthermore, 52.3% of the nurses with 1 to 5 years of ICU work time, 61% of the nurses with 6 to 10 years of ICU work time, 61.4% of the nurses with 11 to 15 years of ICU work time and 87.5% of the nurses with more than 15 years of ICU work time states that patient visiting hours increase their work load. Statistically this is significant. 57% of the nurses consider the visiting hours as having a negative impact on the nursing care given to the patients. About the appropriate visiting hours, 10% of the nurses state that as long as full isolation precautions are applied there should be no restrictions, 89.2% of the nurses state that time, individual and age restrictions should be applied, and 0.8% of the nurses state that it should be severely restricted and a rigid observation of rules should be applied.

## Conclusions

Although, intensive care nurses state that, patient visiting hours are a source of stress for them, increase their workload and negatively affect the quality of the nursing care most of them, they suggested that visits should be continued with brief interviews.

## Grant Acknowledgment

This research received no specific grant from any funding agency in the public, commercial, or not-for-profit sectors.
